# Deep learning–based CT slice synthesis improves radiomic feature reproducibility and discriminative performance in lung nodule assessment

**DOI:** 10.1186/s13244-026-02338-w

**Published:** 2026-06-19

**Authors:** Hujun Yang, Zhengping Zhang, Lei Tian, Pei Dang, Long Wan, Zhiyuan Zhou, Jingjie Li, Lei Cao, Xiaoyan Yang, Juan Chen

**Affiliations:** 1 Department of Computer Center, General Hospital of Ningxia Medical University, Yinchuan, China; 2 Department of Radiology, General Hospital of Ningxia Medical University, Yinchuan, China; 3 Ningxia Medical University, Yinchuan, China; 4 Department of Key Laboratory of Ningxia Stem Cell and Regenerative Medicine, Institute of Medical Sciences, General Hospital of Ningxia Medical University, Yinchuan, China; 5 Department of Pulmonary and Critical Care Medicine, General Hospital of Ningxia Medical University, Yinchuan, China

**Keywords:** Tomography, X-ray computed, Radiomics, Deep learning, Image processing, computer-assisted

## Abstract

**Objectives:**

To investigate the effect of CT slice thickness on radiomic features (RFs) in terms of reproducibility and discriminative power, and to assess whether a deep learning–based CT slice synthesis (DLS) algorithm can mitigate the adverse effects associated with thick-slice CT.

**Materials and methods:**

This retrospective multicenter study included 506 patients with lung nodules (245 benign, 261 malignant) from two independent cohorts, which were divided into a training set, internal validation set (IVS), and external validation set (EVS). Chest CT reconstructed at 1-mm and 5-mm slice thicknesses was analyzed. A DLS algorithm was applied to convert 5-mm CT into synthetic 1-mm CT. RFs were extracted from all CT types to construct radiomics models. Reproducibility was assessed using the concordance correlation coefficient (CCC) and compared with the Wilcoxon signed-rank test. Discriminative power was evaluated by the area under the receiver operating characteristic curve (AUC) and compared with DeLong’s test.

**Results:**

The CCCs of DLS 1-mm CT were 0.48 ± 0.37 and 0.49 ± 0.37 in Cohort 1 and Cohort 2, respectively, significantly higher than real 5-mm CT (all *p* < 0.001). Most RFs from 5-mm CT lacked reproducibility (CCC ≥ 0.85; 0.9% in both Cohort 1 and Cohort 2), whereas DLS 1-mm CT showed marked improvement (Cohort 1, 27.6%; Cohort 2, 26.9%). The discriminative power of RFs from DLS 1-mm CT was superior to that of 5-mm CT and non-inferior to real 1-mm CT, both in model construction and evaluation.

**Conclusion:**

CT slice thickness substantially influences the reproducibility and discriminative power of RFs, whereas the DLS algorithm effectively mitigates the limitations associated with thick-slice CT.

**Critical relevance statement:**

Deep learning–based CT slice synthesis significantly reduces the slice thickness–related variability in radiomics feature reproducibility and discriminative power, providing a promising methodological approach to improve radiomics standardization and support its clinical translation.

**Key Points:**

- CT slice thickness variability substantially impairs radiomic feature reproducibility and discriminative performance, posing a major barrier to standardized radiomics analysis.
- Across two independent cohorts, deep learning–based slice synthesis mitigated the adverse effects of thick-slice CT on radiomic feature reproducibility and cross-thickness discriminative performance.

**Graphical Abstract:**

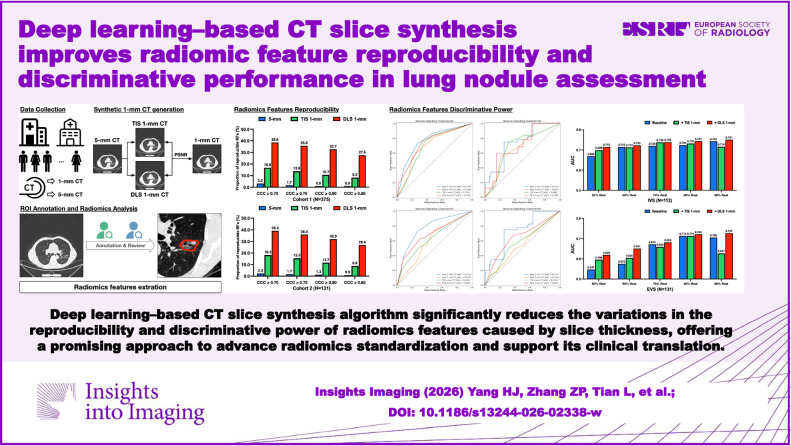

## Introduction

Radiomics applies high-throughput computing to extract quantitative features from medical images, revealing patterns not readily visible to the human eye [1]. It has shown strong potential in precision oncology, supporting diagnosis, risk stratification, and prediction of treatment response [2–4]. Despite growing evidence of its effectiveness, the lack of standardization in the definition and computation of radiomic feature (RF) has limited clinical adoption [5–7]. To address this challenge, Zwanenburg et al [8] proposed the Image Biomarker Standardization Initiative (IBSI), which published a reference manual defining standardized RFs and outlining their calculation procedures.

For CT-based radiomics, a major barrier to clinical translation lies in the poor reproducibility of RFs across varying imaging parameters, raising concerns over the generalizability of findings across centers [9–13]. Among these parameters, slice thickness has been widely recognized as an important factor affecting RF reproducibility. In a phantom-based study, Berenguer et al [11] reported that most RFs (106/177) were sensitive to changes in slice thickness. Similarly, Larue et al [12] found that fewer than half of RFs remained stable when comparing 1.5-mm and 3-mm slice thicknesses. These findings highlight the vulnerability of radiomics to slice thickness variation and have motivated the adoption of image resampling strategies to harmonize voxel resolution prior to feature extraction. Accordingly, the IBSI-recommended processing pipeline incorporates trilinear interpolation to improve RF reproducibility [8].

Beyond conventional interpolation, deep learning–based CT slice synthesis (DLS) has been proposed as a more advanced approach to address slice thickness–related variability. Park et al [13] first reported that DLS could convert thick-slice CT (3–5 mm) into synthetic thin-slice (1-mm) CT and substantially improve RF reproducibility in lung cancer. With ongoing advances in deep learning, multiple DLS algorithms have since been developed [14–17], and prior studies have shown that the visual quality of DLS 1-mm CT is comparable to that of real 1-mm CT [17].

The aim of this multicenter study was to retrospectively evaluate the impact of CT slice thickness on RF reproducibility and discriminative performance and to determine whether the DLS algorithm can mitigate slice thickness–related variability. In addition to assessing RF reproducibility, we examined how slice thickness and slice synthesis influence radiomics-based discriminative power using lung nodule classification as a representative task. Here, discriminative power denotes the ability of radiomic features, when integrated into a classification model, to distinguish malignant from benign lung nodules. By jointly evaluating reproducibility and discrimination, this study aims to provide methodological evidence to support radiomics standardization and improve robustness in heterogeneous clinical datasets.

## Materials and methods

This retrospective study was approved by the institutional review board of each participating center (Number: KYLL-2024-0195, KYLL-2024-0985). The requirement for written informed consent was waived due to the retrospective nature of the study.

### Study participants

**Fig. 1:**
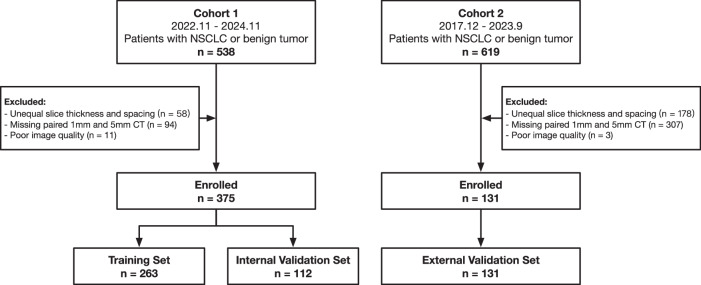
Overview of the patient inclusion process from two independent cohorts and final data distribution

This multicenter retrospective study included patients who underwent chest CT at the General Hospital of Ningxia Medical University (Cohort 1) and the General Hospital of Ningxia Medical University Hospital of Cardio-Cerebral-Vascular-Disease (Cohort 2) between December 2017 and November 2024 (Fig. 1). Cohort 1 comprised 375 patients, with 197 (52.5%) confirmed as non-small cell lung cancer (NSCLC), while the remaining cases were benign. Cohort 2 included 131 patients, with 64 (48.9%) confirmed as NSCLC. All lung nodules were confirmed as benign or malignant based on postoperative histopathology following surgical resection. Patients in Cohort 1 were randomly divided into a training set (*n* = 263) and an internal validation set (IVS, *n* = 112) in a 7:3 ratio. Cohort 2 was used as an external validation set (EVS). All data splitting was performed at the patient level. Exclusion criteria were: (1) inconsistent CT slice thickness and spacing; (2) absence of paired 1-mm and 5-mm CT; and (3) poor image quality. CT scans with severe motion artifacts, prominent streak or beam-hardening artifacts, incomplete lung coverage, or excessive noise that precluded reliable nodule delineation were excluded.

### Image acquisition

Chest CT was performed using either a Brilliance 256 iCT (Philips Healthcare) scanner in Cohort 1 or an Aquilion Multislice Helical CT (TOSHIBA Medical Systems) scanner in Cohort 2, with acquisition parameters of 100–120 kVp, 150–200 mAs, and a 512 × 512 matrix. Scans were acquired during full inspiration in the supine position, covering the region from the lung base to the thoracic inlet. In Cohort 1, reconstruction was performed at 5-mm and 1-mm slice thicknesses using YB and B kernels with iDose level 4, respectively, which are standard lung reconstruction kernels provided by Philips Healthcare. In Cohort 2, reconstruction was performed at 5-mm and 1-mm slice thicknesses using FC18 and FC56 kernels with Adaptive Iterative Dose Reduction in 3D, respectively, which are standard lung reconstruction kernels provided by Toshiba Medical Systems. Kernel-related effects were not independently evaluated, as differences between cohorts reflect real-world multicenter heterogeneity.

### CT slice synthesis

**Fig. 2:**
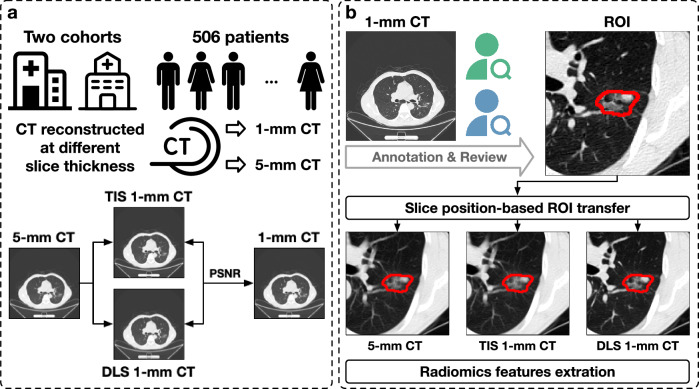
Study flowchart. **a** CT from two independent cohorts was reconstructed at 1-mm and 5-mm slice thickness. Trilinear interpolation (TIS) and deep learning–based CT slice synthesis (DLS) were applied to convert 5-mm CT into synthetic 1-mm CT. The similarity between synthetic and real 1-mm CT was quantitatively evaluated using peak signal-to-noise ratio (PSNR). **b** Lung nodules were manually annotated and reviewed on real 1-mm CT by two radiologists to generate regions of interest (ROIs). To isolate the effects of slice thickness and slice synthesis from annotation variability, the ROIs were propagated to other CT types using a slice position–based strategy. Radiomic features were subsequently extracted from each CT type based on the corresponding ROIs

In this study, a convolutional–transformer hybrid network (CTH-Net) proposed by Yu et al [17], was used to generate synthetic 1-mm CT from real 5-mm CT (Fig. 2a). This model was selected because it demonstrated robust performance in large-scale, multicenter evaluations reported in the original study and provides publicly available code and pretrained parameters, enabling reproducible application to independent datasets without additional model training. In contrast, most prior studies, including the IBSI, employed trilinear interpolation to resample CT resolution. Thus, we generated trilinear interpolation–based synthesis (TIS) 1-mm CT for comparison. For each patient, real 1-mm and 5-mm CT were reconstructed from the same acquisition, and the corresponding TIS 1-mm and DLS 1-mm images were generated from the paired 5-mm CT, ensuring that all four CT types were derived from the same scan and the same patient. Peak signal-to-noise ratios (PSNRs) [18] were used to quantitatively evaluate differences between real and synthetic 1-mm CT.

### Lung nodule segmentation

The region of interest (ROI) was manually segmented using 3D Slicer software (Version 5.2.1, https://www.slicer.org) by a radiologist (P.D., with over 5 years of experience in chest imaging) and subsequently reviewed by a senior radiologist (Z.P.Z., with over 10 years of experience). All radiologists were blinded to clinical information and pathological diagnoses during the process. ROIs were delineated slice by slice to encompass the entire nodule.

As shown in Fig. 2b, the ROIs were manually delineated once on real 1-mm CT images and transferred to the corresponding slices on other CT types based on slice position. Specifically, for each slice in 5-mm CT, the ROI from the real 1-mm CT at the same slice position was directly copied to the corresponding 5-mm slice. If an ROI was present on a 1-mm slice without a matching 5-mm slice position, that ROI was not transferred. For TIS and DLS 1-mm CT, the slice positions were identical to those of the real 1-mm CT. Therefore, the ROI from real 1-mm CT can be completely copied to the TIS and DLS 1-mm CT on a one-to-one basis. To isolate the effects of slice thickness and slice synthesis from annotation-related variability, no additional manual modification or re-segmentation was performed on any of the propagated ROIs across different CT types.

### Radiomics analysis

Radiomics analysis was conducted at the patient level. All CT types derived from the same patient were assigned to the same dataset split (training, IVS, or EVS), ensuring strict patient-level separation. For each CT, 1080 RFs compliant with the IBSI were extracted using PyRadiomics (Version 2.1.0), including 17 first-order statistical features, 73 texture features, and 990 transformation features (270 Laplacian of Gaussian and 720 wavelet). Reproducibility analyses in this study focused on RF agreement across different CT types under an identical ROI. For each RF, the reproducibility was evaluated using concordance correlation coefficients (CCCs), with a threshold of CCC ≥ 0.85 considered reproducible [13].

To assess the RF discriminative power, radiomics-based classification models for NSCLC were developed. Feature preprocessing and selection were performed exclusively on the training set. All features were preprocessed using z-score normalization, with scaling parameters estimated from the training set only. Feature selection was conducted once on the full training set using a three-step procedure. First, a Mann–Whitney U test was conducted to compare each RF between benign and malignant nodules, and features with *p* < 0.05 were retained. Second, Pearson correlation coefficients (r) were computed between all pairs of retained features. For any pair with |r| > 0.85, only the feature with the smaller *p*-value from the first step was retained. Third, a least absolute shrinkage and selection operator (LASSO) regression was applied to select the most informative and non-redundant features. The regularization parameter (λ) in LASSO was determined using ten-fold cross-validation within the training set, with a fixed random seed. The optimal value was selected based on the minimum cross-validated error criterion (λ_min). Rad-score refers to the radiomics-based prediction model constructed using selected radiomic features with a machine learning method, which outputs the predicted probability of malignancy for each lung nodule. To minimize confounding effects from model complexity, multivariate logistic regression was intentionally chosen as a simple and transparent classifier, allowing the analysis to focus on the impact of CT slice thickness and slice synthesis on radiomic feature behavior and downstream model performance rather than on classifier optimization. Based on the above development process, four rad-scores were independently trained from four CT types (real 1-mm, 5-mm, TIS 1-mm, and DLS 1-mm), resulting in Rad-score[1-mm], Rad-score[5-mm], Rad-score[TIS], and Rad-score[DLS], respectively. The optimal λ values were 0.04 for Rad-score[1-mm], 0.05 for Rad-score[5-mm], 0.10 for Rad-score[TIS], and 0.08 for Rad-score[DLS]. Details of the feature selection process for each Rad-score, including the number of retained features at each selection step, the optimal LASSO regularization parameter (λ), and the final selected feature sets, are summarized in Supplementary Table S1.

Two analyses were performed to investigate the effect of slice thickness and slice synthesis on RF discriminative power. In Analysis 1, each well-trained Rad-score was evaluated on the corresponding CT type in the IVS and EVS. Rad-score, together with the normalization parameters, was directly applied to the IVS and EVS to generate predicted probabilities, without any re-selection of features or model refitting. In Analysis 2, well-trained Rad-score[1-mm] was applied to different CT types (5-mm CT, TIS 1-mm CT, and DLS 1-mm CT) in IVS and EVS, using features and normalization parameters corresponding to Rad-score[1-mm]. This analysis was designed to assess generalizability across different types of CT.

In addition, to evaluate the impact of different training data compositions, Rad-scores trained using datasets composed of real 1-mm CT with those supplemented by TIS 1-mm CT or DLS 1-mm CT at varying proportions were compared.

### Statistical analysis

Statistical analyses were performed using SPSS (version 23.0, IBM). Continuous variables following a normal distribution were described as means ± standard deviation (SD); otherwise, they were presented as medians and interquartile ranges (IQR). The categorical variables were described as frequency and percentage (%). The PSNR and CCC were compared using the Wilcoxon signed-rank test. The discriminative power of Rad-score was assessed using receiver operating characteristic (ROC) and area under the ROC curve (AUC). AUCs were compared using the DeLong test. Calibration curves and Brier scores were used to assess probability agreement. The bootstrap method was applied to estimate 95% confidence intervals (CIs). A two-sided *p* < 0.05 was considered statistically significant. Comparisons of AUCs across multiple CT representations were performed for exploratory methodological assessment. Therefore, the corresponding *p*-values should be interpreted as descriptive rather than confirmatory.

## Results

### Patient characteristics

A total of 506 patients (median age, 57.0 [IQR, 50.0–66.0] years) were enrolled, with 245 (48.4%) having benign nodules and 261 (51.6%) having NSCLC. Baseline demographic characteristics are summarized in Table 1. There were no significant differences in the distribution of age (*p* = 0.25), sex (*p* = 0.55), pathological status (*p* = 0.76), nodule location (*p* = 0.83), nodule type (*p* = 0.97), and nodule volume (*p* = 0.44) among the three sets.

**Table 1:** Patient characteristics

	Cohort 1 (*N* = 375)		Cohort 2 (*N* = 131)
	Training set (*N* = 263)	IVS (*N* = 112)	EVS (*N* = 131)
Age (years)	58.0 [50.5, 66.0]	58.5 [51.0, 66.0]	56.0 [49.5, 65.0]
Sex (*N*, %)			
Male	130 (49.4)	60 (53.6)	61 (46.6)
Female	133 (50.6)	52 (46.4)	70 (53.4)
Pathological (*N*, %)			
Malignant	139 (52.9)	58 (51.8)	64 (48.9)
Benign	124 (47.1)	54 (48.2)	67 (51.1)
Location (*N*, %)			
LUL	80 (30.4)	31 (27.7)	31 (23.7)
LLL	42 (16.0)	20 (17.9)	31 (23.7)
RUL	60 (22.8)	29 (25.9)	34 (26.0)
RML	26 (9.9)	11 (9.8)	14 (10.6)
RLL	55 (20.9)	21 (18.7)	21 (16.0)
Nodule type (*N*, %)			
Solid nodules	160 (60.8)	65 (58.0)	77 (58.8)
Part-solid nodules	59 (22.4)	21 (18.8)	28 (21.4)
Ground glass nodules	44 (16.8)	26 (23.2)	26 (19.8)
Nodule volume (cm^3^)	2.2 [0.9, 6.4]	1.8 [0.9, 4.5]	1.7 [0.7, 4.8]

*IVS* internal validation set, *EVS* external validation set, *LUL* left upper lobe, *LLL* left lower lobe, *RUL* right upper lobe, *RML* right middle lobe, *RLL* right lower lobe

### Performance of CT slice synthesis

**Fig. 3:**
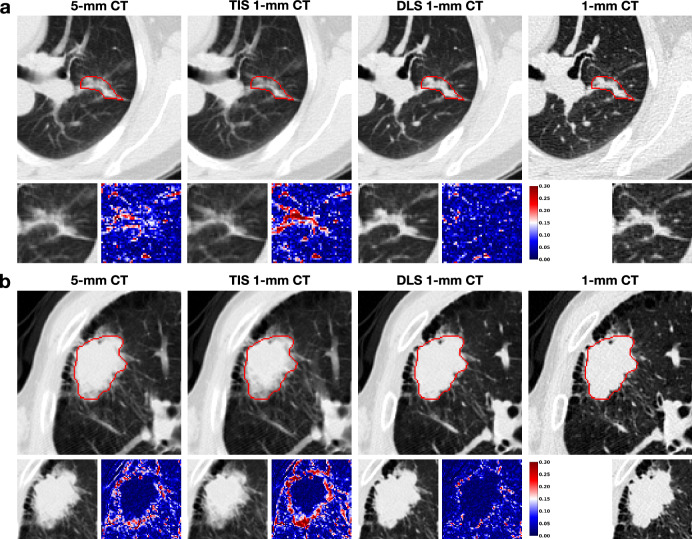
Visual comparisons of different CT images of **a** a 60-year-old woman with a malignant nodule from the internal validation set and **b** a 75-year-old man with a malignant nodule from the external validation set. For each case, corresponding difference maps relative to real 1-mm CT are shown to illustrate voxel-wise intensity deviations. TIS, trilinear interpolation–based synthetic; DLS, deep learning–based CT slice synthesis

Quantitative evaluation demonstrated that DLS 1-mm CT achieved higher PSNR than TIS 1-mm CT in both Cohort 1 (35.90 vs. 33.34, *p* < 0.001) and Cohort 2 (35.37 vs. 33.26, *p* < 0.001). As shown in Table 2, this advantage was consistently observed across the benign group (Cohort 1, 35.76 vs. 33.34; Cohort 2, 35.40 vs. 32.99) and malignant group (Cohort 1, 35.94 vs. 33.33; Cohort 2, 35.27 vs. 33.48), with all comparisons reaching statistical significance (all *p* < 0.001). Subgroup analyses stratified by nodule type and nodule volume further confirmed the superior PSNR performance of DLS compared with TIS (all *p* < 0.001). Illustrative examples are shown in Fig. 3.

**Table 2:** PSNR between different types of CT

	Cohort 1			Cohort 2		
	TIS 1-mm	DLS 1-mm	*p*-value	TIS 1-mm	DLS 1-mm	*p*-value
All	33.34 [32.31, 34.13]	35.90 [34.38, 37.64]	< 0.001	33.26 [32.25, 33.97]	35.37 [34.40, 37.33]	< 0.001
Pathological						
Benign	33.34 [32.22, 34.03]	35.76 [34.28, 37.70]	< 0.001	32.99 [31.93, 33.95]	35.40 [34.40, 37.51]	< 0.001
Malignant	33.33 [32.37, 34.18]	35.94 [34.46, 37.62]	< 0.001	33.48 [32.54, 33.98]	35.27 [34.45, 37.10]	< 0.001
Nodule type						
Solid nodules	33.34 [32.33, 34.03]	35.76 [34.38, 37.42]	< 0.001	33.04 [32.09, 33.99]	35.23 [34.38, 37.11]	< 0.001
Part-solid nodules	33.55 [32.49, 34.35]	36.50 [34.51, 38.09]	< 0.001	33.13 [32.59, 33.90]	35.39 [34.79, 37.61]	< 0.001
Ground glass nodules	33.12 [32.19, 34.16]	35.68 [34.19, 37.71]	< 0.001	33.55 [32.60, 33.94]	35.41 [34.11, 37.37]	< 0.001
Nodule volume						
< 0.5 cm^3^	32.84 [32.00, 34.09]	35.51 [34.05, 38.09]	< 0.001	33.21 [31.54, 33.84]	35.90 [33.91, 37.46]	< 0.001
0.5–14.0 cm^3^	33.37 [32.34, 34.16]	36.21 [34.46, 37.73]	< 0.001	33.40 [32.30, 34.04]	35.39 [34.45, 37.37]	< 0.001
> 14.0 cm^3^	33.29 [32.55, 33.68]	35.12 [34.35, 36.65]	< 0.001	32.87 [32.53, 33.51]	35.00 [34.40, 35.82]	< 0.001

*PSNR* peak signal-to-noise ratio, *TIS* trilinear interpolation–based synthetic, *DLS* deep learning–based CT slice synthesis, *Ref* reference

### Effect of slice thickness on RF

**Fig. 4:**
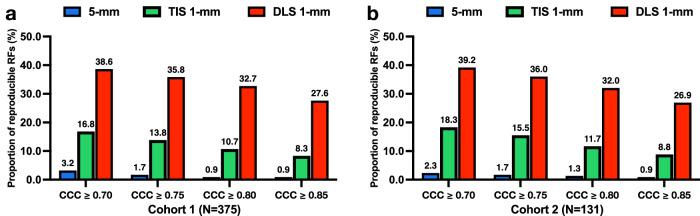
Proportion of reproducible radiomics features (RFs) in various types of CT images on the **a** Cohort 1, and **b** Cohort 2. CCC, concordance correlation coefficient; TIS, trilinear interpolation–based synthetic; DLS, deep learning–based CT slice synthesis

As shown in Table 3, the CCCs between real 1-mm and 5-mm CT were 0.13 [0.04–0.29] in Cohort 1 and 0.13 [0.04–0.25] in Cohort 2. Similar reproducibility patterns were observed across benign and malignant nodules. The proportion of reproducible RFs (CCC ≥ 0.85) was low, with only 0.9% (10/1080) in both Cohort 1 (Fig. 4a) and Cohort 2 (Fig. 4b), detailed in Supplementary Table S3.

**Table 3:** CCC between different types of CT

	All			Benign			Malignant		
	CCC	*p*-value	*p*-value	CCC	*p*-value	*p*-value	CCC	*p*-value	*p*-value
**Cohort 1**		**5-mm**	**TIS 1-mm**		**5-mm**	**TIS 1-mm**		**5-mm**	**TIS 1-mm**
5-mm	0.13 [0.04, 0.29]	Ref		0.13 [0.04, 0.30]	Ref		0.10 [0.03, 0.28]	Ref	
TIS 1-mm	0.19 [0.00, 0.60]	< 0.001	Ref	0.19 [0.01, 0.57]	< 0.001	Ref	0.18 [0.00, 0.60]	< 0.001	Ref
DLS 1-mm	0.47 [0.09, 0.87]	< 0.001	< 0.001	0.49 [0.11, 0.87]	< 0.001	< 0.001	0.40 [0.04, 0.86]	< 0.001	< 0.001
**Cohort 2**		**5-mm**	**TIS 1-mm**		**5-mm**	**TIS 1-mm**		**5-mm**	**TIS 1-mm**
5-mm	0.13 [0.04, 0.25]	Ref		0.12 [0.04, 0.28]	Ref		0.11 [0.02, 0.26]	Ref	
TIS 1-mm	0.17 [0.00, 0.57]	< 0.001	Ref	0.14 [0.01, 0.51]	< 0.001	Ref	0.21 [0.00, 0.63]	< 0.001	Ref
DLS 1-mm	0.52 [0.10, 0.86]	< 0.001	< 0.001	0.50 [0.10, 0.84]	< 0.001	< 0.001	0.44 [0.06, 0.87]	< 0.001	< 0.001

*CCC* concordance correlation coefficient, *Ref* reference, *TIS* trilinear interpolation–based synthetic, *DLS* deep learning–based CT slice synthesis

The effect of slice thickness on discriminative power was summarized in Table 4. When Rad-score[1-mm] was applied to real 1-mm CT, the AUCs for the IVS and EVS were 0.775 and 0.737, respectively. In contrast, Rad-score[5-mm] applied to 5-mm CT yielded lower AUCs of 0.704 (*p* = 0.77) in the IVS and 0.665 (*p* = 0.73) in the EVS. Notably, applying Rad-score[1-mm] to 5-mm CT resulted in a significant performance decline, with AUCs of 0.611 (*p* = 0.009) in the IVS and 0.545 (*p* = 0.002) in the EVS.

**Table 4:** Radiomics models performance analysis

Train data	Test data	IVS (*N* = 112)			EVS (*N* = 131)		
		AUC [95% CI]	*p*-value	*p*-value	AUC [95% CI]	*p*-value	*p*-value
**Analysis 1**			**Real 1-mm**	**Real 5-mm**		**Real 1-mm**	**Real 5-mm**
Real 1 mm	Real 1 mm	0.775 [0.680, 0.856]	Ref		0.737 [0.644, 0.817]	Ref	
Real 5 mm	Real 5 mm	0.704 [0.597, 0.792]	0.77	Ref	0.665 [0.577, 0.755]	0.73	Ref
TIS 1 mm	TIS 1 mm	0.703 [0.609, 0.792]	0.67	> 0.99	0.690 [0.600, 0.787]	> 0.99	> 0.99
DLS 1-mm	DLS 1-mm	0.743 [0.648, 0.830]	> 0.99	> 0.99	0.708 [0.621, 0.796]	> 0.99	> 0.99
**Analysis 2**			**Real 1-mm**	**Real 5-mm**		**Real 1-mm**	**Real 5-mm**
Real 1 mm	Real 1 mm	0.775 [0.680, 0.856]	Ref		0.737 [0.644, 0.817]	Ref	
Real 1 mm	Real 5 mm	0.611 [0.507, 0.712]	0.009*	Ref	0.545 [0.451, 0.644]	0.002^a^	Ref
Real 1 mm	TIS 1 mm	0.636 [0.542, 0.743]	0.06	> 0.99	0.578 [0.477, 0.668]	0.01^a^	> 0.99
Real 1 mm	DLS 1-mm	0.691 [0.593, 0.785]	0.33	0.55	0.653 [0.560, 0.743]	0.28	0.17

*IVS* internal validation set, *EVS* external validation set, *AUC* area under receiver operating characteristic curve, *CI* confidence interval, *TIS* trilinear interpolation–based synthetic, *DLS* deep learning–based CT slice synthesis, *Ref* reference

^a^ Statistically significant difference at a significance level of *p* < 0.05

### Reproducibility of RF after CT slice synthesis

As shown in Table 3, both TIS and DLS improved RF reproducibility in Cohort 1 (TIS 1-mm, 0.19 [0.00–0.60], *p* < 0.001; DLS 1-mm, 0.47 [0.09–0.87], *p* < 0.001) and Cohort 2 (TIS 1-mm, 0.17 [0.00–0.57], *p* < 0.001; DLS 1-mm, 0.52 [0.10–0.86], *p* < 0.001), with DLS yielding a greater improvements than TIS in both cohorts (all *p* < 0.001). Subgroup analyses stratified by pathological status (Table 3), nodule type (Supplementary Table S3) and nodule volume (Supplementary Table S4) consistently demonstrated the superior RF reproducibility of DLS compared with TIS (all *p* < 0.001). Feature-class–level analysis revealed that transformation and texture features were substantially more sensitive to slice thickness than first-order features, and benefited most from DLS (Supplementary Table S5).

Consistent with the observed increase in CCCs, the proportion of reproducible RFs also improved (Fig. 4 and Supplementary Table S2). In Cohort 1, 8.3% (90/1080) of TIS 1-mm RFs and 27.6% (298/1080) of DLS 1-mm RFs met the reproducibility threshold; whereas the corresponding proportions in Cohort 2 were 8.8% (95/1080) and 26.9% (291/1080), respectively.

### Discriminative power of RF after CT slice synthesis

As shown in Table 4, when the training and testing data consisted of the same type of CT, the AUCs of both Rad-score[TIS] and Rad-score[DLS] were lower than Rad-score[1-mm], but higher than Rad-score[5-mm]. Rad-score[DLS] consistently outperformed Rad-score[TIS] in both IVS (0.743 vs. 0.703; Fig. 4a) and EVS (0.708 vs. 0.690; Fig. 4b).

**Fig. 5:**
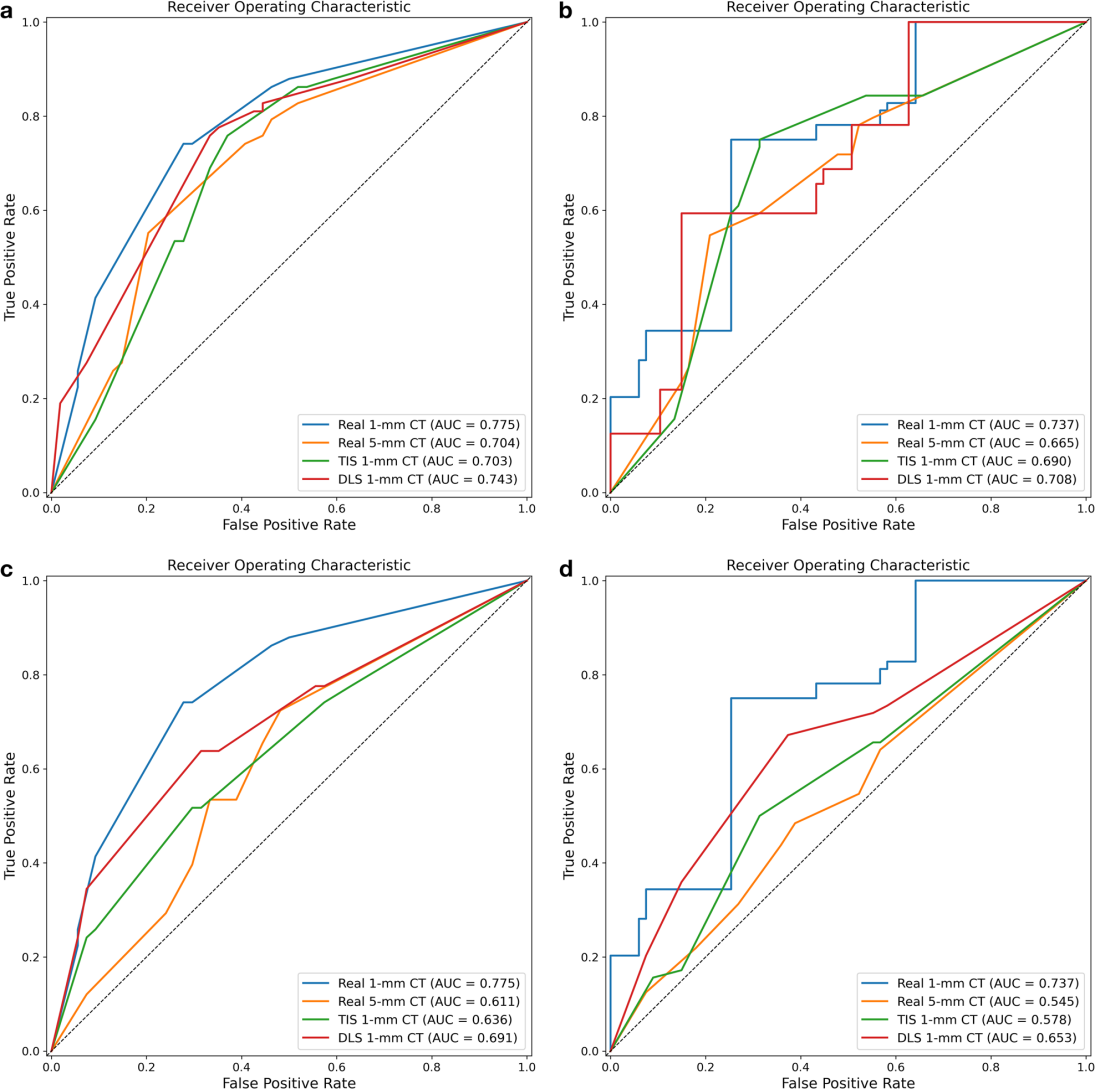
Receiver operating characteristic (ROC) curves of radiomics models in the internal validation set (IVS) and external validation set (EVS). ROCs for radiomics models trained and tested using the same type of CT on **a** IVS and **b** EVS. ROCs for models trained using real 1-mm CT and tested on different types of CT on **c** IVS and **d** EVS. TIS, trilinear interpolation–based synthetic; DLS, deep learning–based CT slice synthesis

When Rad-score[1-mm] was applied to TIS 1-mm CT, the AUC showed little improvement over 5-mm CT, and a significant decline compared to real 1-mm CT was observed in EVS (0.578 vs. 0.737, *p* = 0.01; Fig. 5d). In contrast, applying Rad-score[1-mm] to DLS 1-mm CT yielded AUCs comparable to those obtained from real 1-mm CT, with values of 0.691 (*p* = 0.33) in the IVS (Fig. 5c) and 0.653 (*p* = 0.28) in the EVS (Fig. 5d). Calibration analysis was performed to assess probability agreement across different CT types (Supplementary Fig. S1). In the IVS, Rad-score[1-mm] evaluated on real 1-mm CT showed the best calibration, whereas substantial miscalibration was observed when applying the same model to real 5-mm CT. In the EVS, increased calibration variability was observed across all CT types. DLS 1-mm CT consistently improved calibration relative to real 5-mm CT and TIS 1-mm CT.

**Fig. 6:**
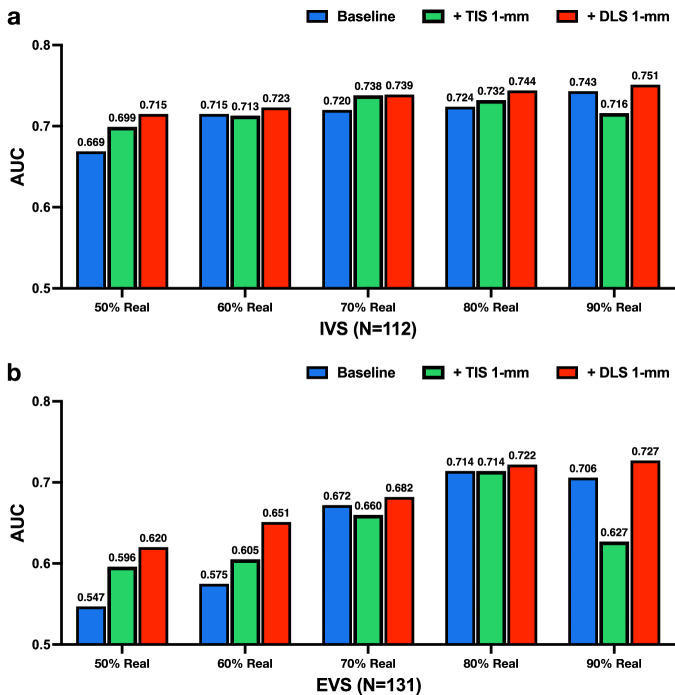
AUCs of radiomics models trained with different data compositions in the **a** internal validation set and **b** external validation set. AUC, area under the receiver operating characteristic curve; TIS, trilinear interpolation–based synthetic; DLS, deep learning–based CT slice synthesis

The discriminative power of Rad-scores developed using mixing CT types at varying proportions was shown in Fig. 6. The baseline Rad-score trained solely on real 1-mm CT showed an upward trend in AUC on both IVS and EVS as the training sample size increased. Adding TIS 1-mm CT to the training set resulted in occasional AUC improvements, but the performance was inconsistent and could impair generalization (Fig. 6b). Across all mixing proportions, Rad-scores trained with additional DLS 1-mm CT consistently achieved the highest AUCs in both validation cohorts, detailed in Supplementary Table S5.

## Discussion

Slice thickness influences CT performance across multiple levels, from visual-level tasks such as lung nodule detection [19, 20] to feature-level quantitative analyses such as radiomics [9–13]. In this multicenter study, we investigated the impact of CT slice thickness on radiomics feature (RF) reproducibility and discriminative power and further assessed whether conventional trilinear interpolation or deep learning–based CT slice synthesis (DLS) could mitigate these effects.

Most RFs are highly sensitive to CT acquisition and reconstruction settings, with slice thickness identified as one of the most influential factors for reproducibility [21], even with the latest photon-counting detector CT [10]. Consistent with previous studies [11–13], we observed that RF reproducibility between 1-mm and 5-mm CT was poor, with only 0.9% of features meeting the reproducibility threshold, reinforcing concerns regarding the generalizability of CT radiomics across heterogeneous protocols. While trilinear interpolation (TIS) is widely adopted in radiomics workflows [8, 22, 23], our results indicate that its ability to resolve slice thickness–related variability remains limited. Unlike trilinear interpolation, which performs linear voxel averaging along the slice direction, DLS learns a nonlinear mapping that exploits contextual information and anatomical priors to infer fine-scale structures. This enables improved preservation of local intensity variation and spatial heterogeneity, which are critical for radiomic feature stability. Park et al [13] first introduced the use of DLS for CT interpolation, increasing the proportion of reproducible RFs from 1.0% to 17.4% for 5-mm CT. In this study, a state-of-the-art open-source DLS algorithm [17] was applied to synthesize 1-mm CT from 5-mm CT. Both participating cohorts of this study can be regarded as independent evaluation datasets for the DLS algorithm, since the pretrained DLS network was applied without retraining or fine-tuning to data from both cohorts. DLS significantly improved RF reproducibility, increasing the median CCC from 0.13 to approximately 0.5, and the proportion of reproducible RFs increased from less than 10% to approximately 27%. However, the absolute reproducibility remained moderate, and most features still did not meet the predefined reproducibility threshold after DLS. Nevertheless, the direction and magnitude of improvement were consistent across centers with different scanners, suggesting that DLS provides a robust, although incomplete, mitigation of slice thickness–related variability in multicenter settings.

Beyond reproducibility, the influence of slice thickness on RF discriminative power has been explored mainly through comparisons between models built on CT with different slice thicknesses. Using lung nodule as a representative task, existing evidence remains inconsistent. In a single-center study, Park et al [24] reported that slice thickness did not significantly affect the performance of radiomics models for predicting disease-free survival in NSCLC patients. Similarly, Zhang et al [25] found that slice thickness had minimal impact on RF’s diagnostic performance when differentiating malignant from benign lung nodules. In contrast, Xu et al [26] demonstrated that the effect of slice thickness on RF’s discriminative power was dependent on lesion size. A concise overview of representative studies examining the impact of slice thickness on radiomics is provided in Supplementary Table S7. In our study, we first compared models developed from different CT types to quantify the impact of slice thickness on discriminative power and cross-thickness generalizability. The radiomics models trained on real 1-mm CT showed a marked performance decline when applied to 5-mm CT, indicating poor cross-thickness generalizability. We further evaluated whether slice synthesis could mitigate this degradation. TIS provided limited mitigation, whereas RFs derived from DLS 1-mm CT effectively preserved performance, achieving results comparable to real 1-mm CT in both internal and external validations. The observation that different CT types yielded non-identical feature sets highlights the sensitivity of RF selection to image representation. Importantly, DLS mitigated the downstream impact of this variability by enabling consistent model performance across slice thicknesses, indicating improved robustness of the radiomics pipeline. Calibration analysis further illustrates that DLS has less calibration distortion than real 5-mm CT and TIS.

In real-world clinical datasets, inconsistent slice thickness across examinations is common, and a frequently adopted strategy is to convert thick-slice CT into thin-slice CT and combine the data for model training [8]. Our comparative analysis directly evaluated this practice. Models trained on CT with mixed slice thickness showed that incorporating DLS 1-mm CT consistently improved model performance, while adding trilinear interpolated 1-mm CT yielded inconsistent results. These findings suggest that although slice thickness alters RF distribution, its impact on discriminative power can be mitigated through DLS, supporting its utility for harmonizing heterogeneous CT datasets in radiomics pipeline. Together, these findings indicate that DLS is not merely an image interpolation technique, but also a transferable preprocessing strategy that stabilizes radiomic feature behavior across heterogeneous slice thicknesses. By improving both feature reproducibility and cross-thickness discriminative consistency without model retraining, DLS offers a practical solution for mitigating slice thickness–induced variability in multicenter radiomics studies.

Several limitations should be acknowledged. First, this study involved a limited number of cohorts and focused on binary classification between NSCLC and benign lung nodules. Although improved RF reproducibility and preserved discriminative performance are important prerequisites for clinical translation, this study was not designed to establish clinical validity. Outcome-oriented clinical prediction tasks, such as survival or treatment response modeling, were not evaluated, and the impact of slice thickness–sensitive features in real-world clinical prediction settings warrants further investigation in larger, more diverse multicenter cohorts. Second, while this study focused on slice thickness, the 1-mm and 5-mm CT images within each cohort were reconstructed using different kernels according to the corresponding clinical protocols. Therefore, the observed differences may reflect the combined effects of slice thickness and reconstruction kernel, rather than slice thickness alone. Other acquisition and reconstruction parameters may also influence RF behavior and were not systematically evaluated. Third, inter-rater agreement for ROI delineation was not formally quantified. In this study, ROIs were manually delineated on real 1-mm CT by one radiologist and subsequently reviewed and corrected by a senior radiologist to obtain consensus annotations. This consensus-based strategy was adopted to minimize annotation-related variability and to isolate the effects of slice thickness and CT slice synthesis on radiomic feature behavior. In addition, because the ROIs for real 5-mm CT were generated using a slice position–based transfer strategy, slice-level ROIs present on real 1-mm CT without a matching 5-mm slice position were not transferred, which may have introduced differences in analyzed ROI volume across CT types. Although this approach preserved positional correspondence and avoided interpolation of annotations, segmentation variability and ROI volume mismatch may still have influenced radiomic feature reproducibility in real-world settings. Future studies incorporating independently drawn ROIs from multiple readers and more refined cross-thickness ROI alignment are needed to comprehensively evaluate their combined effects on radiomic reproducibility. Fourth, when evaluating cross–CT-type generalizability by applying Rad-score[1-mm] to other CT types, we assessed calibration to characterize probability consistency under domain shift but did not perform explicit recalibration, as the goal of this study was methodological comparison of CT types rather than optimization for clinical deployment. Fifth, shape features were excluded to reduce confounding effects from slice thickness–dependent segmentation variability, as all ROIs were referenced from real 1-mm CT. While this design choice facilitated focused evaluation of slice thickness effects on intensity- and texture-based features, it limits conclusions regarding shape features, which remain clinically relevant. Finally, while trilinear interpolation was used as the IBSI-recommended baseline, it represents only one interpolation method and may not be optimal. Thus, it may be premature to conclude that the DLS algorithm outperforms all conventional interpolation techniques. In addition, as a data-driven image transformation method, the performance of the DLS algorithm may be influenced by domain shift, including variations in scanner type, acquisition protocol, and reconstruction kernel. Although the pretrained DLS algorithm demonstrated consistent improvements across two independent cohorts in this study, its robustness under more heterogeneous imaging conditions warrants further investigation. Moreover, compared with conventional interpolation, the DLS algorithm introduces additional computational cost and requires dedicated hardware resources, such as GPUs, which may limit its scalability or routine deployment in resource-constrained settings.

In conclusion, this multicenter study demonstrates that CT slice thickness substantially influences the reproducibility and discriminative power of radiomic features. Compared with IBSI-recommended trilinear interpolation, deep learning–based CT slice synthesis (DLS) more effectively improves feature stability and downstream model performance across diverse cohorts. These findings support the integration of DLS into the radiomics pipeline as a promising approach to standardize image preprocessing and improve the generalizability of radiomics in clinical practice.

## Supplementary information

ELECTRONIC SUPPLEMENTARY MATERIAL

## Data Availability

The data used or analyzed during the current study are available from the corresponding author on reasonable request.

## Abbreviations

AUC: Area under the receiver operating characteristic curve
CCC: Concordance correlation coefficient
CI: Confidence interval
DLS: Deep learning–based CT slice synthesis
EVS: External validation set
IVS: Internal validation set
LASSO: Least absolute shrinkage and selection operator
NSCLC: Non-small cell lung cancer
PSNR: Peak signal-to-noise ratio
RF: Radiomic feature
ROC: Receiver operating characteristic
ROI: Regions of interest
TIS: Trilinear interpolation–based synthesis
