# Supplementary material for: Deep learning–based CT slice synthesis improves radiomic feature reproducibility and discriminative performance in lung nodule assessment
Source: Insights Imaging. 2026 Jun 19;17:163. doi: 10.1186/s13244-026-02338-w (PMC13282418; doi:10.1186/s13244-026-02338-w)
Supplement: Supplementary file 1 — ELECTRONIC SUPPLEMENTARY MATERIAL [file 13244_2026_2338_MOESM1_ESM.pdf]

Deep Learning–Based CT Slice Synthesis Improves Radiomic Feature Reproducibility and Discriminative Performance in Lung Nodule Assessment

ELECTRONIC SUPPLEMENTARY MATERIAL

Insights Imaging (2026) Yang HJ, Zhang ZP, Tian L, et al.

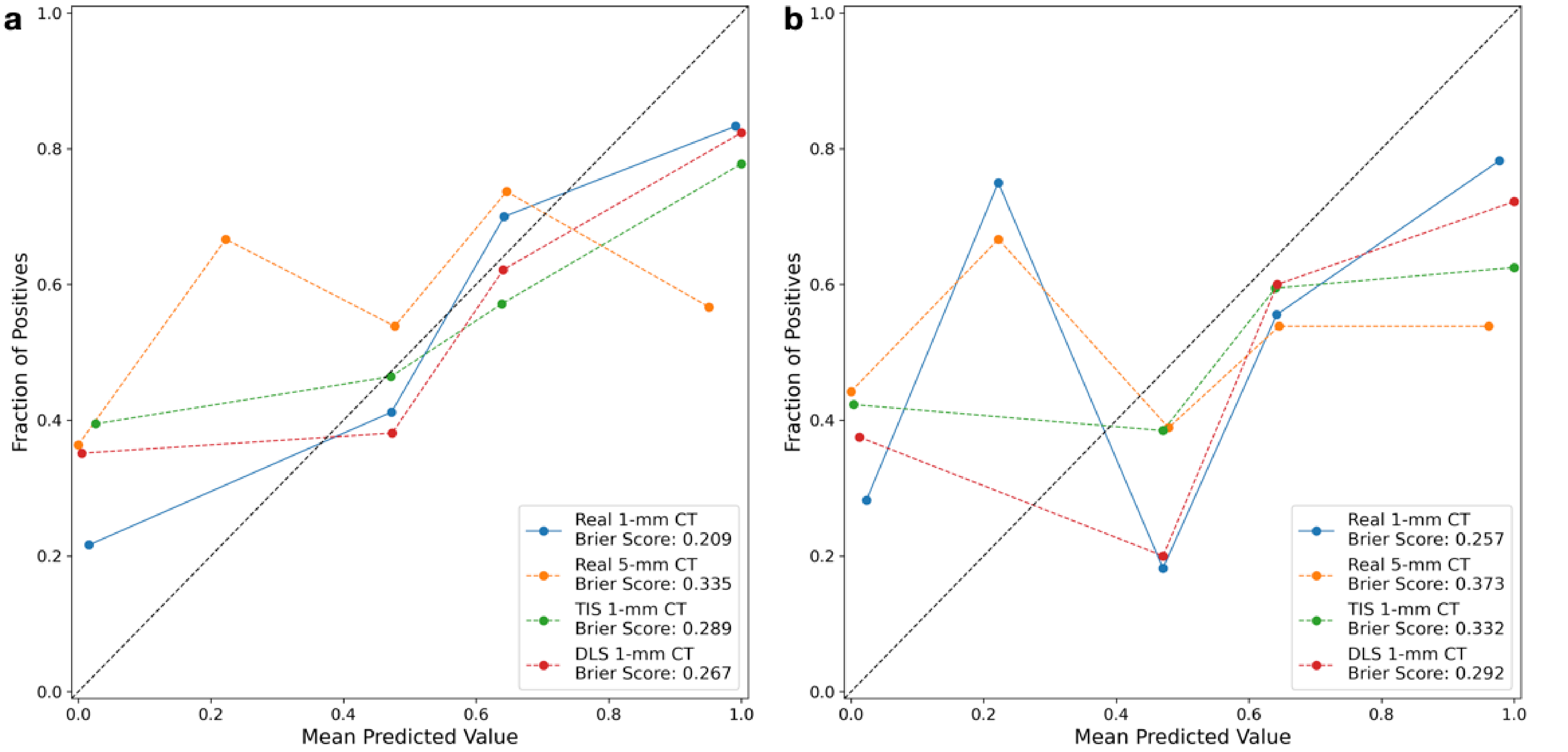

Figure S1. Calibration curves of radiomics model trained using real 1-mm CT and tested on different types of CT in the (a) internal validation set, and (b) external validation set. TIS, trilinear interpolation–based synthetic; DLS, deep learning–based CT slice synthesis.

Table S1. Feature selection process

	Feature Number			LASSO regularization parameter	Features
	Significance testing	Correlation filtering	LASSO		
Rad-score [1-mm]	665	45	8	0.04	firstorder_Maximum<br>log-sigma-1-0-mm-3D_firstorder_90Percentile<br>log-sigma-1-0-mm-3D_firstorder_Mean<br>log-sigma-1-0-mm-3D_firstorder_Minimum<br>wavelet-HHL_firstorder_Maximum<br>wavelet-HHL_ngtdm_Busyness<br>wavelet-LHL_ngtdm_Strength<br>wavelet-LLL_glcm_JointEntropy
Rad-score [5-mm]	165	39	10	0.05	original_glcm_JointEntropy<br>original_ngtdm_Strength<br>log-sigma-1-0-mm-3D_glszm_GrayLevelNonUniformity<br>log-sigma-2-0-mm-3D_gldm_LargeDependenceEmphasis<br>log-sigma-2-0-mm-3D_glszm_LargeAreaHighGrayLevelEmphasis<br>wavelet-HHH_gldm_LargeDependenceEmphasis<br>wavelet-HHH_ngtdm_Busyness<br>wavelet-HHL_ngtdm_Busyness<br>wavelet-LHL_ngtdm_Strength<br>wavelet-LLL_firstorder_Energy

Rad-score [TIS]	327	70	12	0.10	log-sigma-1-5-mm-3D_ngtdm_Strength<br>log-sigma-2-0-mm-3D_glszm_GrayLevelVariance<br>log-sigma-2-0-mm-3D_glszm_ZoneVariance<br>wavelet-HHH_firstorder_Kurtosis<br>wavelet-HLH_firstorder_Kurtosis<br>wavelet-HLH_ngtdm_Busyness<br>wavelet-LHH_ngtdm_Busyness<br>wavelet-LLH_firstorder_Kurtosis<br>wavelet-LLH_glcm_Contrast<br>wavelet-LLH_gldm_LargeDependenceLowGrayLevelEmphasis<br>wavelet-LLL_firstorder_Energy<br>wavelet-LLL_glcm_JointEntropy
Rad-score [DLS]	305	47	7	0.08	original_glszm_ZoneEntropy<br>log-sigma-2-0-mm-3D_glcm_Correlation<br>log-sigma-2-0-mm-3D_ngtdm_Contrast<br>wavelet-LHH_glcm_Imc2<br>wavelet-LHL_glcm_MCC<br>wavelet-LLH_glcm_Correlation<br>wavelet-LLH_glcm_Imc1

NOTE: LASSO, least absolute shrinkage and selection operator; TIS, trilinear interpolation–based synthetic; DLS, deep learning–based CT slice synthesis

Table S2. Number of RFs meeting reproducibility criteria according to different thresholds.

Image Type	CCC ≥ 0.70	CCC ≥ 0.75	CCC ≥ 0.80	CCC ≥ 0.85
**Cohort 1**				
**5-mm**	35 (3.2)	18 (1.7)	10 (0.9)	10 (0.9)
**TIS 1-mm**	181 (16.8)	149 (13.8)	116 (10.7)	90 (8.3)
**DLS 1-mm**	417 (38.6)	387 (35.8)	353 (32.7)	298 (27.6)
**Cohort 2**				
**5-mm**	25 (2.3)	18 (1.7)	14 (1.3)	10 (0.9)
**TIS 1-mm**	198 (18.3)	167 (15.5)	126 (11.7)	95 (8.8)
**DLS 1-mm**	423 (39.2)	389 (36.0)	346 (32.0)	291 (26.9)

NOTE: RF, radiomics feature; CCC, concordance correlation coefficient; TIS, trilinear interpolation–based synthetic; DLS, deep learning–based CT slice synthesis.

Table S3. CCC between different types of CT.

	Solid nodules			Part-solid nodules			Ground glass nodules		
	**CCC**	***p***	***p***	**CCC**	***p***	***p***	**CCC**	***p***	***p***
**Cohort 1**		**5-mm**	**TIS 1-mm**		**5-mm**	**TIS 1-mm**		**5-mm**	**TIS 1-mm**
**5-mm**	0.11 [0.02, 0.26]	**Ref**		0.09 [0.03, 0.25]	**Ref**		0.12 [0.04, 0.25]	**Ref**	
**TIS 1-mm**	0.22 [0.00, 0.61]	< 0.001	**Ref**	0.10 [0.00, 0.37]	0.002	**Ref**	0.13 [0.00, 0.39]	< 0.001	**Ref**
**DLS 1-mm**	0.47 [0.06, 0.87]	< 0.001	< 0.001	0.35 [0.04, 0.79]	< 0.001	< 0.001	0.41 [0.06, 0.82]	< 0.001	< 0.001
**Cohort 2**		**5-mm**	**TIS 1-mm**		**5-mm**	**TIS 1-mm**		**5-mm**	**TIS 1-mm**
**5-mm**	0.10 [0.03, 0.24]	**Ref**		0.05 [0.00, 0.18]	**Ref**		0.10 [0.03, 0.20]	**Ref**	
**TIS 1-mm**	0.15 [0.00, 0.55]	< 0.001	**Ref**	0.10 [0.00, 0.38]	< 0.001	**Ref**	0.12 [0.00, 0.39]	< 0.001	**Ref**
**DLS 1-mm**	0.47 [0.06, 0.86]	< 0.001	< 0.001	0.43 [0.06, 0.83]	< 0.001	< 0.001	0.39 [0.05, 0.80]	< 0.001	< 0.001

NOTE: CCC, concordance correlation coefficient; Ref, Reference; TIS, trilinear interpolation–based synthetic; DLS, deep learning–based CT slice synthesis.

Table S4. CCC between different types of CT.

	Volume < 0.5 cm^3			0.5 cm^3 ≤ Volume ≤ 14.0 cm^3			Volume > 14.0 cm^3		
	**CCC**	***p***	***p***	**CCC**	***p***	***p***	**CCC**	***p***	***p***
**Cohort 1**		**5-mm**	**TIS 1-mm**		**5-mm**	**TIS 1-mm**		**5-mm**	**TIS 1-mm**
**5-mm**	0.06 [0.01, 0.16]	**Ref**		0.09 [0.03, 0.26]	**Ref**		0.06 [0.00, 0.23]	**Ref**	
**TIS 1-mm**	0.09 [0.00, 0.29]	< 0.001	**Ref**	0.16 [0.00, 0.48]	< 0.001	**Ref**	0.18 [0.00, 0.63]	< 0.001	**Ref**
**DLS 1-mm**	0.34 [0.07, 0.78]	< 0.001	< 0.001	0.44 [0.06, 0.84]	< 0.001	< 0.001	0.27 [0.02, 0.85]	< 0.001	< 0.001
**Cohort 2**		**5-mm**	**TIS 1-mm**		**5-mm**	**TIS 1-mm**		**5-mm**	**TIS 1-mm**
**5-mm**	0.07 [0.03, 0.17]	**Ref**		0.08 [0.02, 0.20]	**Ref**		0.06 [0.01, 0.23]	**Ref**	
**TIS 1-mm**	0.09 [0.00, 0.22]	0.006	**Ref**	0.11 [0.00, 0.41]	< 0.001	**Ref**	0.17 [0.00, 0.67]	< 0.001	**Ref**
**DLS 1-mm**	0.37 [0.06, 0.73]	< 0.001	< 0.001	0.40 [0.04, 0.81]	< 0.001	< 0.001	0.30 [0.02, 0.90]	< 0.001	< 0.001

NOTE: CCC, concordance correlation coefficient; Ref, Reference; TIS, trilinear interpolation–based synthetic; DLS, deep learning–based CT slice synthesis.

Table S5. CCC between different types of CT.

	First-order Features (N=17)			Texture Features (N=73)			Transformation Features (N=990)		
	**CCC**	***p***	***p***	**CCC**	***p***	***p***	**CCC**	***p***	***p***
**Cohort 1**		**5-mm**	**TIS 1-mm**		**5-mm**	**TIS 1-mm**		**5-mm**	**TIS 1-mm**
**5-mm**	0.70 [0.65, 0.90]	**Ref**		0.34 [0.18, 0.52]	**Ref**		0.12 [0.03, 0.25]	**Ref**	
**TIS 1-mm**	0.66 [0.53, 0.89]	0.99	**Ref**	0.43 [0.26, 0.59]	0.14	**Ref**	0.15 [0.00, 0.59]	< 0.001	**Ref**
**DLS 1-mm**	0.95 [0.89, 0.98]	< 0.001	< 0.001	0.71 [0.57, 0.85]	< 0.001	< 0.001	0.40 [0.07, 0.86]	< 0.001	< 0.001
**Cohort 2**		**5-mm**	**TIS 1-mm**		**5-mm**	**TIS 1-mm**		**5-mm**	**TIS 1-mm**
**5-mm**	0.63 [0.53, 0.89]	**Ref**		0.30 [0.18, 0.45]	**Ref**		0.11 [0.03, 0.23]	**Ref**	
**TIS 1-mm**	0.60 [0.41, 0.90]	0.24	**Ref**	0.44 [0.21, 0.58]	0.09	**Ref**	0.13 [0.00, 0.56]	< 0.001	**Ref**
**DLS 1-mm**	0.94 [0.87, 0.99]	< 0.001	< 0.001	0.71 [0.56, 0.85]	< 0.001	< 0.001	0.45 [0.08, 0.86]	< 0.001	< 0.001

NOTE: CCC, concordance correlation coefficient; Ref, Reference; TIS, trilinear interpolation–based synthetic; DLS, deep learning–based CT slice synthesis.

Table S6. Radiomics models' performance with different ratios of real and synthetic data.

Ratio	IVS (N=112)		EVS (N=131)	
	**AUC [95% CI]**	***p***	**AUC [95% CI]**	***p***
**50% Real**				
Baseline (N=131)	0.669 [0.569, 0.761]	**Ref**	0.547 [0.453, 0.638]	**Ref**
+ TIS 1-mm	0.699 [0.601, 0.786]	> 0.99	0.596 [0.504, 0.684]	0.86
+ DLS 1-mm	0.715 [0.622, 0.799]	0.87	0.620 [0.531, 0.708]	0.62
**60% Real**				
Baseline (N=157)	0.715 [0.626, 0.796]	**Ref**	0.575 [0.483, 0.664]	**Ref**
+ TIS 1-mm	0.713 [0.609, 0.802]	> 0.99	0.605 [0.518, 0.691]	0.89
+ DLS 1-mm	0.723 [0.629, 0.807]	> 0.99	0.651 [0.558, 0.736]	0.63
**70% Real**				
Baseline (N=184)	0.720 [0.628, 0.811]	**Ref**	0.672 [0.581, 0.759]	**Ref**
+ TIS 1-mm	0.738 [0.642, 0.821]	> 0.99	0.660 [0.570, 0.745]	> 0.99
+ DLS 1-mm	0.739 [0.638, 0.829]	> 0.99	0.682 [0.587, 0.772]	> 0.99
**80% Real**				
Baseline (N=210)	0.724 [0.635, 0.809]	**Ref**	0.714 [0.632, 0.789]	**Ref**
+ TIS 1-mm	0.732 [0.630, 0.822]	> 0.99	0.714 [0.618, 0.798]	> 0.99
+ DLS 1-mm	0.744 [0.652, 0.833]	> 0.99	0.722 [0.634, 0.815]	> 0.99
**90% Real**				
Baseline (N=236)	0.743 [0.654, 0.832]	**Ref**	0.706 [0.620, 0.791]	**Ref**
+ TIS 1-mm	0.716 [0.613, 0.817]	> 0.99	0.627 [0.539, 0.724]	0.34
+ DLS 1-mm	0.751 [0.655, 0.839]	> 0.99	0.727 [0.636, 0.803]	> 0.99

NOTE: IVS, internal validation set; EVS, external validation set; AUC, area under receiver operating characteristic curve; CI, Confidence interval; TIS, trilinear interpolation–based synthetic; DLS, deep learning–based CT slice synthesis; Ref, Reference.

Table S7. Representative studies evaluating the impact of CT slice thickness on radiomics

**Focus of analysis**	**Study (Year)**	**Data type**	**Main findings**
Reproducibility	Berenguer et al. (2018) [1]	CT<br>Phantom study	71 of 177 radiomic features were reproducible when extracted from CT images acquired using different slice thicknesses (2mm, 3mm, 5mm, and 8mm slice thicknesses)
	Meyer et al. (2019) [2]	CT<br>Human study	12.3% [13/106] radiomic features were reproducible when extracted from CT images acquired using different slice thickness (1mm, 3mm, 5mm)
	Emaminejad (2021) [3]	CT<br>Human study	Slice thickness demonstrated the largest impact on radiomic feature values where only one to five features were reproducible out of 226 radiomic features analyzed.
	Peng et al. (2022) [4]	CT<br>Phantom study	23.55% [305/1295] radiomic features were reproducible when extracted from CT images acquired using different slice thickness (1mm, 2mm, 5mm)
	Gupta et al. (2023) [5]	CT<br>Human study	Out of 107 radiomic features, 66 (61.6%) exhibited a significant distinction ($p<0.05$) when comparing 2-mm and 5-mm CT, 29 features showed excellent to moderate reproducibility.
	Varghese et al. (2024) [6]	CT<br>Phantom study	The interquartile range (IQR), coefficient of variation (CV) and standard deviation (SD) of first-order radiomics features are assessed by varying slice thickness using scans of a uniform water phantom, anthropomorphic liver phantom, and a human liver in vivo. In general, SD, CV, and IQR improve (decrease) as slice thickness increases.
	Zhou et al (2025) [7]	CT<br>Human study	Radiomic feature stability is significantly affected by CT reconstruction parameters, especially slice thickness. Varying slice thickness alone (1 mm, 3 mm, 5 mm) for the same patient's CT images resulted in 7.0% (97/1394) of features exhibiting stability.
	Zhang et al. (2025) [8]	PCD-CT<br>Phantom study	16.1% [15/93] radiomic features were reproducible when extracted from CT images acquired using different slice thickness (0.4mm, 1mm).

Discrimination	Park et al. (2021) [9]	CT<br>Human study	The performance of radiomics models for predicting disease-free survival (DFS) in non-small-cell lung cancer patients was not significantly affected by CT slice thickness. In corresponding slice thickness datasets, the C-indices of Rad-1, Rad-3, and Rad-5 for prediction of DFS were 0.73, 0.73, and 0.76 in the validation set. Performance of the models was not significantly changed when they were applied to different slice thicknesses data in the validation set (C-index, 0.73–0.76, 0.72–0.73, 0.75–0.76; $p = 0.07–0.92$).
	Xu et al. (2021) [10]	CT<br>Human study	For pulmonary nodules less than 1 cm, CT image acquisition parameters have a significant influence on diagnostic performance of radiomics in predicting malignancy, and a model created using images reconstructed with thin section and a sharp kernel algorithm achieved the best performance. For pulmonary nodules larger than 1 cm, CT reconstruction parameters did not affect diagnostic performance substantially.
	Zhang et al. (2023) [11]	CT<br>Human study	There were 75 benign and 73 malignant pulmonary nodules, with mean diameters of 18.63 and 19.86 mm, respectively. The pulmonary nodules were segmented, and 1409 radiomics features were extracted. Slice thickness did not affect the diagnostic performance.
Deep learning	Park et al (2019) [12]	CT<br>Human study	The mean CCCs for the comparisons of original 1 mm vs. 3 mm, 1 mm vs. 5 mm, and 3 mm vs. 5 mm images were 0.41, 0.27, and 0.65, respectively ($p < 0.001$ for all comparisons). The majority of RFs failed to achieve reproducibility ($CCC \geq 0.85$; 3.6%, 1.0%, and 21.5%, respectively). After applying the CNN-based SR algorithms, the reproducible RFs increased (36.3%, 17.4%, and 36.9%, respectively). The reproducibility of RFs in lung cancer is significantly influenced by CT slice thickness, which can be improved by the CNN-based SR algorithms.
	Yang et al. (2021) [13]	CT<br>Human study	The DL method showed a significantly higher CCC value than interpolation-based methods. For features in the tumor region, compared with the cubic interpolation

			approach, the reproducible features increased from 393 (82%) to 422(88%) for the conversion of 3–1 mm, and from 305(64%) to 353(74%) for the conversion of 5–1 mm. For features in the tumor ring region, the improvement was from 395 (82%) to 431 (90%) and from 290 (60%) to 335 (70%), respectively.

NOTE: This table is intended to summarize methodological characteristics and reported trends, rather than to provide a direct quantitative performance comparison.

Reference

[1] Berenguer R, Pastor-Juan M D R, Canales-Vázquez J, et al. Radiomics of CT features may be nonreproducible and redundant: influence of CT acquisition parameters[J]. Radiology, 2018, 288(2): 407-415.

[2] Meyer M, Ronald J, Vernuccio F, et al. Reproducibility of CT radiomic features within the same patient: influence of radiation dose and CT reconstruction settings[J]. Radiology, 2019, 293(3): 583-591.

[3] Emaminejad N, Wahi-Anwar M W, Kim G H J, et al. Reproducibility of lung nodule radiomic features: Multivariable and univariable investigations that account for interactions between CT acquisition and reconstruction parameters[J]. Medical physics, 2021, 48(6): 2906-2919.

[4] Peng X, Yang S, Zhou L, et al. Repeatability and reproducibility of computed tomography radiomics for pulmonary nodules: a multicenter phantom study[J]. Investigative radiology, 2022, 57(4): 242-253.

[5] Gupta S, Nayak K, Pendem S. Impact of slice thickness on reproducibility of CT radiomic features of lung tumors[J]. F1000Research, 2023, 12: 1319.

[6] Varghese B A, Cen S Y, Jensen K, et al. Investigating the role of imaging factors in the variability of CT-based texture analysis metrics[J]. Journal of Applied Clinical Medical Physics, 2024, 25(4): e14192.

[7] Zhou Q, Lin C, Jiang J, et al. Impact of CT acquisition settings on the stability of radiomic features and the performance of pulmonary nodule classification models[J]. Insights into Imaging, 2026, 17(1): 4.

[8] Zhang H, Lu T, Wang L, et al. Robustness of radiomics within photon-counting detector CT: impact of acquisition and reconstruction factors[J]. European Radiology, 2025: 1-13.

[9] Park S, Lee S M, Kim S, et al. Performance of radiomics models for survival prediction in non-small-cell lung cancer: influence of CT slice thickness[J]. European Radiology, 2021, 31(5): 2856-2865.

[10] Xu Y, Lu L, Sun S H, et al. Effect of CT image acquisition parameters on diagnostic performance of radiomics in predicting malignancy of pulmonary nodules of different

sizes[J]. European radiology, 2022, 32(3): 1517-1527.
[11] Zhang R, Shi J, Liu S, et al. Performance of radiomics models derived from different CT reconstruction parameters for lung cancer risk prediction[J]. BMC Pulmonary Medicine, 2023, 23(1): 132.
[12] Park S, Lee S M, Do K H, et al. Deep learning algorithm for reducing CT slice thickness: effect on reproducibility of radiomic features in lung cancer[J]. Korean journal of radiology, 2019, 20(10): 1431-1440.
[13] Yang P, Xu L, Wan Y, et al. Deep neural network-based approach to improving radiomics analysis reproducibility in liver cancer: effect on image resampling[J]. Physics in Medicine & Biology, 2021, 66(16): 165009.
